# Systemic aspergillosis in a patient with interferon gamma receptor 1 deficiency; a case report

**DOI:** 10.1186/s12887-023-04093-z

**Published:** 2023-06-05

**Authors:** Hossein Esmaeilzadeh, Zahra Chavoshzadeh, Seyed Hesamedin Nabavizadeh, Soheila Alyasin, Ali Amanati, Aida Askarisarvestani

**Affiliations:** 1 Division of Allergy and Clinical Immunology, Department of Pediatrics, School of Medicine, Shiraz University of Medical Sciences, Shiraz, Iran; 2 Allergy Research Center, School of Medicine, Shiraz University of Medical Sciences, Shiraz, Iran; 3 Division of Allergy and Clinical Immunology, Department of Pediatrics, Mofid Hospital, Shahid Beheshti University of Medical Sciences, Tehran, Iran; 4 Division of Infectious Diseases, Department of Pediatrics, School of Medicine, Shiraz University of Medical Sciences, Shiraz, Iran; 5 Clinical Microbiology Research Center, Shiraz University of Medical Sciences, Shiraz, Iran

**Keywords:** IFNGR1D, SH2B3, Aspergillosis

## Abstract

**Background:**

Interferon-gamma receptor deficiency is a heterogeneous spectrum of disease which involves mutations in *IFNGR1*, *IFNGR2* genes, and the downstream signaling proteins such as STAT1. These mutations are associated with immunodeficiency 27 A and 27B, making the patient prone to mycobacterial infections. Patients with this condition are also at increased risk for affliction with viral and bacterial infections, such as with the Herpesviridae family, Listeria, and Salmonella. Moreover, SH2B3 mutation is associated with autoimmune and lymphoproliferative conditions.

**Case presentation:**

the patient was a 19-month-old infant girl who presented with a two-week history of fever. She had near-normal flowcytometry with high IgM and IgE. She had pneumonic infiltration in her chest and right hilar and para-aortic lymphadenopathy. PCR of whole blood for Aspergillus fumigatus came back positive. In her Whole Exome Sequencing she had *IFNGR1* and *SH2B3* mutations.

**Conclusion:**

systemic fungal infections such as Aspergillosis can occur in patients with interferon-gamma receptor one deficiency. This type of immunodeficiency should be considered in treating patients with systemic Aspergillosis.

## Introduction

Interferon-gamma receptor deficiency is a heterogeneous spectrum of disease which involves mutations in *IFNGR1*, *IFNGR2* genes, and the downstream signaling proteins such as *STAT1* [1]. These mutations are associated with immunodeficiency 27 A and 27B, making the patient prone to mycobacterial infections [1–3]. Patients with this condition are also at increased risk for affliction with viral and bacterial infections, such as with the Herpesviridae family, Listeria, and Salmonella [4, 5].

Lack of cytokine production after interferon addition and high serum levels of interferon-gamma are the diagnostic hallmarks of the disease [6]. The inheritance of these conditions might follow autosomal recessive or autosomal dominant modes with complete or partial penetrance [7]. Complete autosomal recessive type of Interferon-gamma receptor one deficiency carries the worst prognosis, while the partial autosomal dominant form has the best prognosis [8, 9]. Hematopoietic Stem Cell Transplantation (HSCT) is the mainstay of treatment in these patients [9].

In this study, we report a case of Interferon gamma receptor deficiency with aspergillosis who has presented with generalized lymphadenopathy.

## Case presentation

The patient was a 19-month-old infant girl who presented with a two-week history of fever. with no sign of cough, respiratory distress, or abdominal tenderness. The patient did not have any history of recurrent infections; however, since the patient’s sibling had passed away at two months old following vaccination with BCG, oral poliovirus vaccine, and hepatitis B vaccine, the possibility of immunodeficiency was considered. The consanguinity history of her parents was positive and the patient’s grandmother suffered from systemic lupus erythematous. She had hepatosplenomegaly and generalized lymphadenopathy in her physical examination. The patient had not received BCG vaccine due to the possibility of hemophagocytic lymphohistiocytosis (HLH). HLH was then ruled out by bone marrow aspiration and biopsy which showed essentially cellular marrow with mild shift to the left and no evidence of hemophagocytosis. Chest and abdominopelvic CT scans were taken which showed multiple lymphadenopathies in hilar and para-aortic regions as well as ground-glass opacities suggestive of pneumonic infiltration in the lower lobes of both lungs. Immunodeficiency workup was done for the patient and the results of her laboratory studies are summarized in Table 1. She had near-normal flowcytometry with high IgM and IgE. Moreover, since she had eosinophilia in her complete blood count, fungal infections were considered. PCR of whole blood for Aspergillus fumigatus came back positive and the whole blood PCR for Mycobacterium came back negative. The Whole Exome Sequencing of the patient showed autosomal recessive *IFNGR1* (NM_000416:exon6) mutation of Chr6-137522134 T-c.745delA:p.1249Ffs*11 which was confirmed by Sanger sequencing method. Also, the parents of the patient underwent whole exome sequencing and they were heterozygote for the same mutation; thus, showing the autosomal recessive nature of this disease.

The patient also had a homozygote mutation in *SH2B3* mutation; however, this mutation was not confirmed by Sanger sequencing method due to financial constraints.

**Table 1:** Clinical lab data of the patient at 19 months old

Lab data	result	Reference range
WBC (/mm^3^)	26,100	4000–11,000
Neutrophils (%)	57	-
Lymphocytes (%)	24	-
Eosinophils (%)	**16**	-
Monocytes (%)	2	-
Basophils (%)	1	-
ESR (mm/hr)	85 (H)	< 20
CRP (?)	122 (H)	< 6
Serum IgM (g/L)	4.10 (H)	0.19–1.5
Serum IgG (g/L)	10.1	4.53–9.16
Serum IgA (g/L)	0.70	0.22–1.18
Serum IgE (IU/mL)	133.0 (H)	< 50
Fibrinogen (mg/dL)	375	200–550
Uric acid (mg/dL)	5.1	3.6–7.7
Triglycerides (mg/dL)	265 (H)	< 150
Total cholesterol (mg/dL)	137	< 200
LDH (IU/L)	388	120–300
Ferritin (ng/mL)	473.5 (H)	10.9–117
CH50 (U/mL)	72.0	50–150
DHR (%)	95	≥ 95
CD3 (%) (absolute count)	71.96 (5617.71)	53–84 (2500–5500)
CD4 (%) (absolute count)	49.21 (3841.68)	35–61 (1600–4000)
CD8 (%) (absolute count)	17.70 (1381.78)	12–28 (560–1700)
CD4/CD8 ratio	2.78	1–3
CD7 (%) (absolute count)	77.78 (6073.62)	57–87 (2670–6100)
CD19 (%) (absolute count)	17.38 (1356.80)	6–32 (300–2000)
CD20 (%) (absolute count)	17.40 (1358.36)	6–32 (300–2000)
CD16 (%)	11.49	5–19
CD56 (%)	6.54	5–19
CD16/56 (%) (absolute count)	5.99 (467.62)	4–18 (170–1100)
Interferon gamma (pg/mL)	65	< 18

The patient then received two weeks of intravenous Voriconazole 4 mg/Kg every 12 h, Vancomycin 10 mg/Kg every six hours, and Meropenem 40 mg/Kg every eight hours. She was discharged with oral Voriconazole 4 mg/Kg every 12 h for three months. In her follow up session after one month she had no signs and symptoms and her lab data was as follows in Table 2.

**Table 2:** Follow-up lab data of the patient at 20 months old

Lab data	result	Reference range
WBC (/mm^3^)	20,400	4000–11,000
Neutrophils (%)	24	-
Lymphocytes (%)	50	-
Eosinophils (%)	24	-
Monocytes (%)	1	-
Basophils (%)	1	-
Hb (g/dL)	11.1	12–14
Platelets (/mm^3^)	682,000	150,000-450,000
ESR (mm/hr)	13	< 20
CRP (mg/dL)	20	< 6

She was planned to have hematopoietic stem cell transplantation and HLA typing was done for her and her family members; however, no matched donor was found. Prophylactic antibacterial, antiviral, and antifungal antibiotics were started for her. Moreover, due to the mutation in *SH2B3* she was put on Rituximab 375 mg/m^2^/dose once every two weeks; however, after three doses of Rituximab, she developed severe bloody diarrhea which in further follow up turned out to be Salmonellosis which she was previously prone to due to IFNGRD. Rituximab was discontinued for the patient but she developed septic shock and is currently under treatment for this condition.

## Discussion

In this article, we described a case with interferon gamma receptor 1 deficiency who came with generalized lymphadenopathy and had systemic aspergillosis.

IFNGR1 gene mutations lead to autosomal recessive immunodeficiency type 27 A and autosomal dominant immunodeficiency type 27B as a part of a heterogenous group of diseases called Mendelian Susceptibility to Mycobacterial Disease (MSMD) [10]. The patients are also susceptible to low-virulence intracellular pathogens such as viruses, Histoplasma, and Toxoplasma [11].

Upon connection of interferon gamma to its receptors, various cytokines are released from macrophages and dendritic cells to induce the microbicidal effects of the leukocytes [1]. Multiple case reports have described systemic infections with non-tuberculous mycobacteria and intracellular microorganisms in patients with IFNGR1D [12–16]. However, no systemic infection with Aspergillus fumigatus in this context had been reported.

SH2B3 is another gene that was mutated in this patient. This gene is responsible for stages in the process of negative regulation of hematopoiesis [17]. Consequently, mutated SH2B3 can result in hypercellularity of lymph nodes and spleen. This phenomenon can predispose the patients to hematologic malignancies such as lymphoma this mechanism of action explains the reason behind the patient’s generalized lymphadenopathy. Due to this reason, the patients with this condition better undergo routine investigations to rule out malignancies.

Mutations in *SH2B3* gene have also been associated with autoimmune diseases such as type 1 diabetes mellitus, celiac disease, systemic lupus erythematous, rheumatoid arthritis, and multiple sclerosis [18–21]. Consequently, the probability of autoimmunity should also be considered in this patient.

## Conclusion

systemic fungal infections such as Aspergillosis can occur in patients with interferon-gamma receptor one deficiency. This type of immunodeficiency should be considered in treating patients with systemic Aspergillosis.

## Data Availability

Not applicable.

## References

[1] Bustamante J, Boisson-Dupuis S, Abel L, Casanova JL. Mendelian susceptibility to mycobacterial disease: genetic, immunological, and clinical features of inborn errors of IFN-γ immunity. Semin Immunol. 2014 Dec;26(6):454–70. 10.1016/j.smim.2014.09.008. Epub 2014 Oct 26. PMID: 25453225; PMCID: PMC4357480. 10.1016/j.smim.2014.09.008 PMC4357480 25453225

[2] Dorman SE, Picard C, Lammas D, Heyne K, van Dissel JT, Baretto R, Rosenzweig SD, Newport M, Levin M, Roesler J, Kumararatne D, Casanova JL, Holland SM. Clinical features of dominant and recessive interferon gamma receptor 1 deficiencies. Lancet. 2004 Dec 11–17;364(9451):2113-21. doi: 10.1016/S0140-6736(04)17552-1. PMID: 15589309. 10.1016/S0140-6736(04)17552-1 15589309

[3] Koh WJ, Kwon OJ, Hwang JH, Kim EJ. Partial interferon-gamma receptor deficiency and disseminated tuberculosis. Int J Tuberc Lung Dis. 2005 Apr;9(4):469; author reply 469 – 70. PMID: 15830757. 15830757

[4] Dorman SE, Uzel G, Roesler J, Bradley JS, Bastian J, Billman G, King S, Filie A, Schermerhorn J, Holland SM. Viral infections in interferon-gamma receptor deficiency. J Pediatr. 1999 Nov;135(5):640–3. 10.1016/s0022-3476(99)70064-8. PMID: 10547254; PMCID: PMC7095028. 10.1016/S0022-3476(99)70064-8 PMC7095028 10547254

[5] Roesler J, Kofink B, Wendisch J, Heyden S, Paul D, Friedrich W, Casanova JL, Leupold W, Gahr M, Rösen-Wolff A. Listeria monocytogenes and recurrent mycobacterial infections in a child with complete interferon-gamma-receptor (IFNgammaR1) deficiency: mutational analysis and evaluation of therapeutic options. Exp Hematol. 1999 Sep;27(9):1368-74. doi: 10.1016/s0301-472x(99)00077-6. PMID: 10480427. 10.1016/s0301-472x(99)00077-6 10480427

[6] Lee PP, Lao-Araya M, Yang J, Chan KW, Ma H, Pei LC, Kui L, Mao H, Yang W, Zhao X, Trakultivakorn M, Lau YL. Application of Flow Cytometry in the Diagnostics Pipeline of primary Immunodeficiencies underlying disseminated *Talaromyces marneffei* infection in HIV-Negative children. Front Immunol 2019 Sep 13;10:2189. doi: 10.3389/fimmu.2019.02189. PMID: 31572394; PMCID: PMC6753679. 10.3389/fimmu.2019.02189 PMC6753679 31572394

[7] Lamhamedi S, Jouanguy E, Altare F, Roesler J, Casanova JL. Interferon-gamma receptor deficiency: relationship between genotype, environment, and phenotype (Review). Int J Mol Med. 1998 Feb;1(2):415-8. doi: 10.3892/ijmm.1.2.415. PMID: 9852244. 10.3892/ijmm.1.2.415 9852244

[8] Auld B, Urquhart D, Walsh M, Nourse C, Harris MA. Blurring the lines in interferon {gamma} receptor deficiency: an infant with near-fatal airway disease. Pediatrics. 2011 May;127(5):e1352-5. doi: 10.1542/peds.2010-0387. Epub 2011 Apr 4. PMID: 21464185. 10.1542/peds.2010-0387 21464185

[9] Zhang SY, Boisson-Dupuis S, Chapgier A, Yang K, Bustamante J, Puel A, Picard C, Abel L, Jouanguy E, Casanova JL. Inborn errors of interferon (IFN)-mediated immunity in humans: insights into the respective roles of IFN-alpha/beta, IFN-gamma, and IFN-lambda in host defense. Immunol Rev. 2008 Dec;226:29–40. doi: 10.1111/j.1600-065X.2008.00698.x. PMID: 19161414. 10.1111/j.1600-065X.2008.00698.x 19161414

[10] van de Vosse E, van Dissel JT, Ottenhoff TH. Genetic deficiencies of innate immune signalling in human infectious disease. Lancet Infect Dis. 2009 Nov;9(11):688 – 98. doi: 10.1016/S1473-3099(09)70255-5. PMID: 19850227. 10.1016/S1473-3099(09)70255-5 19850227

[11] van de Vosse E, van Dissel JT. IFN-γR1 defects: Mutation update and description of the IFNGR1 variation database. Hum Mutat. 2017 Oct;38(10):1286–1296. doi: 10.1002/humu.23302. Epub 2017 Aug 3. PMID: 28744922. 10.1002/humu.23302 28744922

[12] Bossi G, Errichiello E, Zuffardi O, Marone P, Monzillo V, Barbarini D, Vergori A, Bassi LA, Rispoli GA, De Amici M, Zecca M. Disseminated *Mycobacterium Avium* infection in a child with Complete Interferon-γ receptor 1 Deficiency due to compound heterozygosis of *IFNGR1* for a Subpolymorphic Copy Number Variation and a Novel splice-site variant. J Pediatr Genet 2020 Sep;9(3):186–92. doi: 10.1055/s-0039-1700803. Epub 2019 Nov 4. PMID: 32714620; PMCID: PMC7375849. 10.1055/s-0039-1700803 PMC7375849 32714620

[13] Obinata K, Lee T, Niizuma T, Kinoshita K, Shimizu T, Hoshina T, Sasaki Y, Hara T. Two cases of partial dominant interferon-γ receptor 1 deficiency that presented with different clinical courses of bacille Calmette-Guérin multiple osteomyelitis. J Infect Chemother. 2013 Aug;19(4):757 – 60. doi: 10.1007/s10156-012-0493-5. Epub 2012 Oct 11. PMID: 23053508. 10.1007/s10156-012-0493-5 23053508

[14] Prucha M, Grombirikova H, Zdrahal P, Bloomfield M, Parackova Z, Freiberger T. Mendelian susceptibility to Mycobacterial Disease: the First Case of a diagnosed adult patient in the Czech Republic. Case Rep Immunol 2020 Dec 19;2020:8836685. doi: 10.1155/2020/8836685. PMID: 33414972; PMCID: PMC7769627. 10.1155/2020/8836685 PMC7769627 33414972

[15] Brummer E. Human defenses against Cryptococcus neoformans: an update. Mycopathologia. 1998–1999;143(3):121-5. doi: 10.1023/a:1006905331276. PMID: 10353206. 10.1023/a:1006905331276 10353206

[16] Malmgaard L. Induction and regulation of IFNs during viral infections. J Interferon Cytokine Res. 2004 Aug;24(8):439 – 54. doi: 10.1089/1079990041689665. PMID: 15320958. 10.1089/1079990041689665 15320958

[17] Huang X, Li Y, Tanaka K, Moore KG, Hayashi JI. Cloning and characterization of Lnk, a signal transduction protein that links T-cell receptor activation signal to phospholipase C gamma 1, Grb2, and phosphatidylinositol 3-kinase. Proc Natl Acad Sci U S A 1995 Dec 5;92(25):11618–22. doi: 10.1073/pnas.92.25.11618. PMID: 8524815; PMCID: PMC40453. 10.1073/pnas.92.25.11618 PMC40453 8524815

[18] Hunt KA, Zhernakova A, Turner G, Heap GA, Franke L, Bruinenberg M, Romanos J, Dinesen LC, Ryan AW, Panesar D, Gwilliam R, Takeuchi F, McLaren WM, Holmes GK, Howdle PD, Walters JR, Sanders DS, Playford RJ, Trynka G, Mulder CJ, Mearin ML, Verbeek WH, Trimble V, Stevens FM, O’Morain C, Kennedy NP, Kelleher D, Pennington DJ, Strachan DP, McArdle WL, Mein CA, Wapenaar MC, Deloukas P, McGinnis R, McManus R, Wijmenga C, van Heel DA. Newly identified genetic risk variants for celiac disease related to the immune response. Nat Genet 2008 Apr;40(4):395–402. doi: 10.1038/ng.102. Epub 2008 Mar 2. PMID: 18311140; PMCID: PMC2673512. 10.1038/ng.102 PMC2673512 18311140

[19] Gateva V, Sandling JK, Hom G, Taylor KE, Chung SA, Sun X, Ortmann W, Kosoy R, Ferreira RC, Nordmark G, Gunnarsson I, Svenungsson E, Padyukov L, Sturfelt G, Jönsen A, Bengtsson AA, Rantapää-Dahlqvist S, Baechler EC, Brown EE, Alarcón GS, Edberg JC, Ramsey-Goldman R, McGwin G Jr, Reveille JD, Vilá LM, Kimberly RP, Manzi S, Petri MA, Lee A, Gregersen PK, Seldin MF, Rönnblom L, Criswell LA, Syvänen AC, Behrens TW, Graham RR. A large-scale replication study identifies TNIP1, PRDM1, JAZF1, UHRF1BP1 and IL10 as risk loci for systemic lupus erythematosus. Nat Genet. 2009 Nov;41(11):1228–33. 10.1038/ng.468. Epub 2009 Oct 18. PMID: 19838195; PMCID: PMC2925843. 10.1038/ng.468 PMC2925843 19838195

[20] Coenen MJ, Trynka G, Heskamp S, Franke B, van Diemen CC, Smolonska J, van Leeuwen M, Brouwer E, Boezen MH, Postma DS, Platteel M, Zanen P, Lammers JW, Groen HJ, Mali WP, Mulder CJ, Tack GJ, Verbeek WH, Wolters VM, Houwen RH, Mearin ML, van Heel DA, Radstake TR, van Riel PL, Wijmenga C, Barrera P, Zhernakova A. Common and different genetic background for rheumatoid arthritis and coeliac disease. Hum Mol Genet. 2009 Nov 1;18(21):4195 – 203. doi: 10.1093/hmg/ddp365. Epub 2009 Jul 31. PMID: 19648290. 10.1093/hmg/ddp365 19648290

[21] Alcina, A & Vandenbroeck, Koen & Otaegui, David & Saiz, A & Gonzalez, Juan & Fernández,Oscar & Lopez Cavanillas, Maria & Cenit, Maria & Arroyo, Rafael & Alloza, Iraide &Barcina, María & Rodríguez-Antigüedad, Alfredo & Leyva, Laura & Izquierdo, Guillermo& Lucas, Miguel & Fedetz, Maria & Pinto-Medel, M & Olascoaga, Javier & Blanco, Yolanda& Matesanz, Fuencisla. (2010). The autoimmune disease-associated KIF5A, CD226 and SH2B3 gene variants confer susceptibility for multiple sclerosis. Genes and immunity.11. 439 – 45. 10.1038/gene.2010.30. 10.1038/gene.2010.30 20508602

